# Hydrogen diffusion and its electrical properties variation as a function of the IGZO stacking structure

**DOI:** 10.1038/s41598-022-24212-7

**Published:** 2022-11-17

**Authors:** Hee Yeon Noh, Woo-Geun Lee, Haripriya G. R., Jung-Hwa Cha, June-Seo Kim, Won Seok Yun, Myoung-Jae Lee, Hyeon-Jun Lee

**Affiliations:** 1 Division of Nanotechnology, Daegu Gyeongbuk Institute of Science and Technology (DGIST), Daegu, 42988 Republic of Korea; 2 Development Team, Samsung Display, Gyeonggi, 17113 Republic of Korea

**Keywords:** Materials for devices, Electronic devices

## Abstract

The oxygen vacancies and hydrogen in oxide semiconductors are regarded as the primary sources of charge carriers and various studies have investigated the effect of hydrogen on the properties of oxide semiconductors. However, the carrier generation mechanism between hydrogen and oxygen vacancies in an *a*-IGZO semiconductor has not yet been clearly examined. In this study we investigated the effect of hydrogen and the variation mechanisms of electrical properties of a thin film supplied with hydrogen from the passivation layer. SiO_x_ and SiN_x_, which are used as passivation or gate insulator layers in the semiconductor process, respectively, were placed on the top or bottom of an *a*-IGZO semiconductor to determine the amount of hydrogen penetrating the *a*-IGZO active layer. The hydrogen diffusion depth was sufficiently deep to affect the entire thin semiconductor layer. A large amount of hydrogen in SiN_x_ directly affects the electrical resistivity of *a*-IGZO semiconductor, whereas in SiO_x_, it induces a different behavior from that in SiN_x_, such as inducing an oxygen reaction and O–H bond behavior change at the interface of an *a*-IGZO semiconductor. Moreover, the change in electrical resistivity owing to the contribution of free electrons could be varied based on the bonding method of hydrogen and oxygen.

## Introduction

Metal oxide semiconductor thin-film transistor (TFTs), especially indium-gallium-zinc-oxide based ones, have received considerable interest since Hosono group reported the first amorphous IGZO (*a*-IGZO) based TFT in 2004^1^. The *a*-IGZO based TFTs have been extensively studied due to their excellent performance attributes such as a high field-effect mobility, good electrical uniformity, large-area stability, and its endurance^2–6^. Out of the candidate materials available for oxide-based TFTs, *a*-IGZO is considered as the most suitable switching layer for application in advanced display backplanes^7,8^. Unlike other covalent bond-based semiconductors, such as Si-based semiconductors, oxide semiconductors have an ionic bonding structure with unique electrical properties, which is primarily caused by the changes in the oxygen vacancies in their structure^9,10^. Because oxygen vacancies and hydrogen in oxide semiconductors are regarded as the primary sources of charge carriers, hydrogen treatments effectively control the electrical performance of *a*-IGZO thin films^11^, and various studies have investigated the effect of hydrogen on the properties of oxide semiconductors^12–14^. Oxygen vacancies can be controlled through various methods, such as controlling the processing conditions (i.e., gas partial pressure^15^, input power^16^, and temperatures during deposition^17^), altering the material composition, and administering post-treatment (i.e., thermal annealing^18^, high pressure^19^, and hydrogen plasma^20^). Owing to the high electronegativity of oxygen in the ionic bonding of oxide semiconductors, hydrogen incorporation in oxide semiconductors is unavoidable^21^. Hydrogen in an oxide semiconductor acts as a shallow donor by ionizing and bonding with oxygen to form O–H bonds^22–24^. The hydrogen in the active layer can originate from the ambient atmosphere (a large amount of hydrogen is present in air), which results in unintentional *n*-doping. In addition, hydrogen can easily penetrate into the active layer by the formation of the gate insulator, the deposition of the passivation layer, and the heat treatment process. However, the carrier generation mechanism between hydrogen and oxygen vacancies in an *a*-IGZO thin films has not yet been clearly examined, and further investigation is necessary.

In this paper, we report the role of hydrogen in *a*-IGZO semiconductors, which can affect their electrical properties. The dynamic increase or decrease in the electrical conductivity of an *a*-IGZO semiconductor was studied by including SiO_x_ and SiN_x_, which were used as the passivation or gate insulating layers in the semiconductor process. The changes observed in the electrical properties of the semiconductor were verified to be closely related to the amount of hydrogen present in the two insulators. The correlation between the hydrogen content inside the *a*-IGZO semiconductor and the binding state of the surrounding elements was also discussed.

## Methods

**Figure 1:**
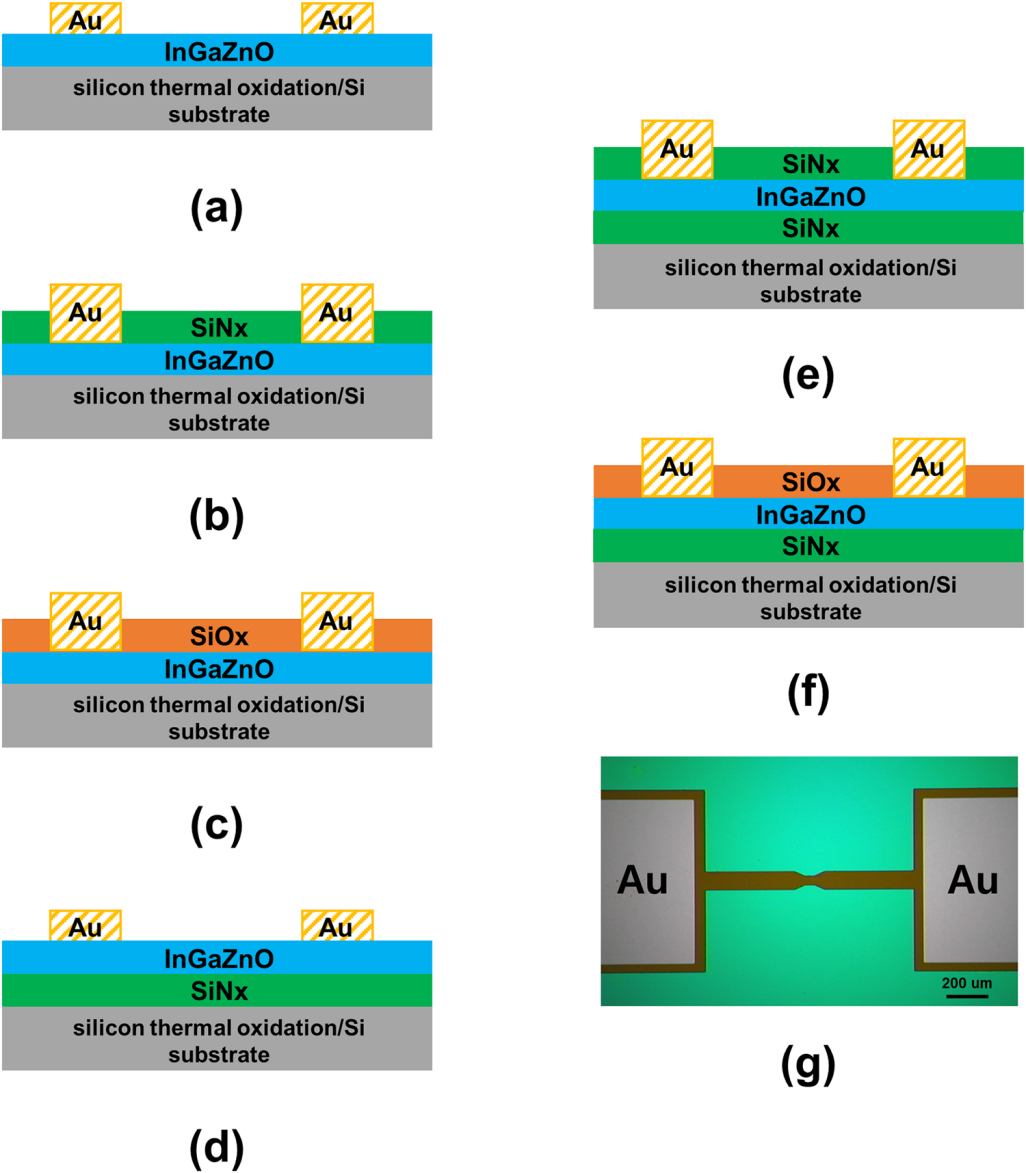
(**a**)–(**f**) Cross-sectional schematics of *a*-IGZO structures covered with SiO_x_ and/or SiN_x_, (**g**) microscopy image of *a*-IGZO resistor pattern.

Schematics of the cross-sectional views of *a*-IGZO films covered with SiO_x_ or SiN_x_ are shown in Fig. 1; the films were prepared under the same deposition conditions and using the same fabrication process used for the *a*-IGZO active layer. A 100 nm thick *a*-IGZO active layer was deposited by sputtering a ceramic target with InO_x_:GaO_x_:ZnO_x_ at a molar ratio of 1:1:1.5 at room temperature on a silicon thermal oxidation/Si substrate. To measure the electrical properties, a resistor pattern was formed in the *a*-IGZO active layer via photolithography. Notably, in this study, the SiN_x_ and SiO_x_ layers were used as hydrogen diffusion source layers. Typically, SiN_x_ contains a large amount of hydrogen. In contrast, SiO_x_ contains a relatively low amount of hydrogen. Both SiN_x_ and SiO_x_ were used as insulator or passivation layers in the conventional device. SiO_x_ and SiN_x_ films of 50 nm thickness were deposited via plasma-enhanced chemical vapor deposition (PECVD) at 350 °C. The source gas flow, pressure, and radio frequency power were SiH_4_/NH_3_ = 15/30 sccm, 1 torr, and 150 W for SiN_x_, respectively, and SiH_4_/N_2_O = 30/2000 sccm, 1 torr, and 100 W for SiO_x_, respectively. Six different thin-film structures were prepared to evaluate the effect of hydrogen according to the position of the hydrogen source: conventional *a*-IGZO (Fig. 1a), SiN_x_/*a*-IGZO (Fig. 1b), SiO_x_/*a*-IGZO (Fig. 1c), *a*-IGZO/SiN_x_ (Fig. 1d), SiN_x_/*a*-IGZO/SiN_x_ (Fig. 1e), and SiO_x_/*a*-IGZO/SiN_x_ (Fig. 1f). To minimize the influence of the contact resistance, the gold via contact electrodes of 100 nm thickness were formed by sputtering. After the fabrication process, the fabricated stacks were annealed at 350 °C for 1 h in air. A Keithley 2636 B dual-source meter was used to measure the electrical properties of the *a*-IGZO thin films with width/length of 50 *um* /100 *um*. Time-of-flight secondary ion mass spectrometry (TOF–SIMS) and X-ray photoelectron spectroscopy (XPS) were used to measure and analyze the amount of hydrogen in the films and chemical composition depth profiles of the films, respectively. The channel length and critical dimension of the fabricated device were measured using TRM 200 (thermal reflective microscope, Nanoscope systems).

## Results and discussion

**Figure 2:**
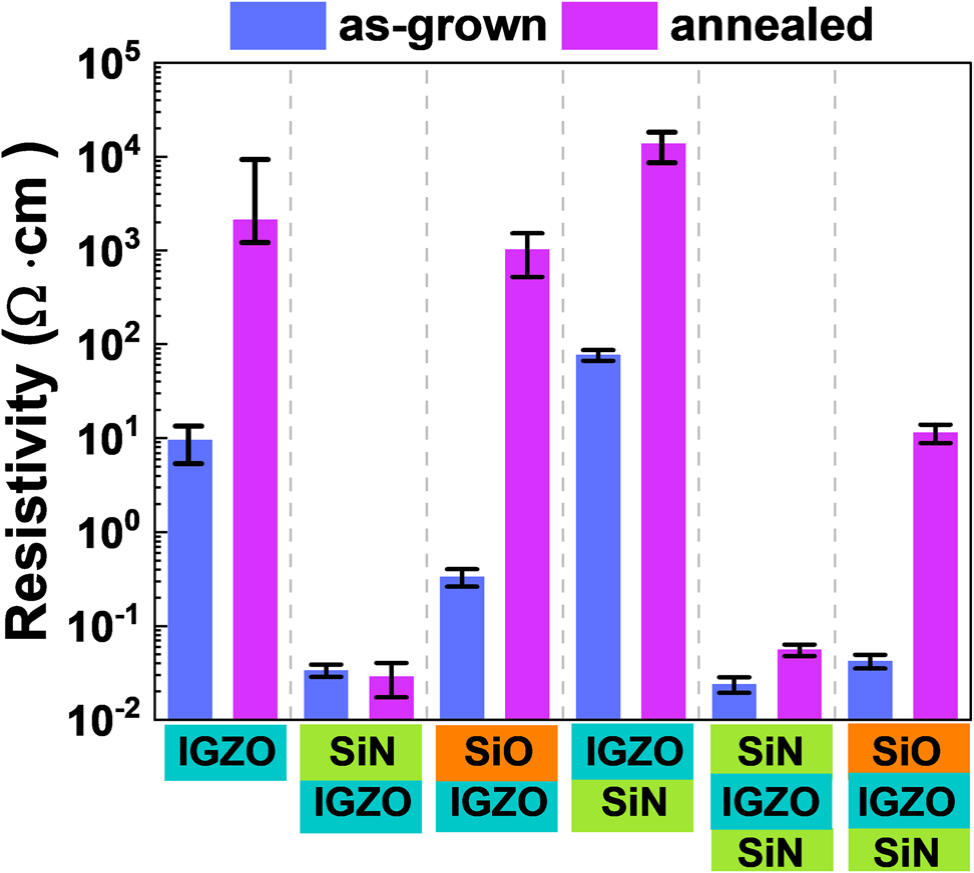
Electrical properties of as-grown and annealed *a*-IGZO films with different structures of passivation or gate insulator layers.

Electrical measurements at the two terminals were performed using a semiconductor probing system. The electrical resistivity measured between the source and drain without a gate electrode is shown in a bar chart (Fig. 2) for the as-grown and annealed *a*-IGZO films, respectively. The resistivity of the as-grown *a*-IGZO film was approximately 10 Ω∙cm, and the resistivity was confirmed to increase by two orders of magnitude after the annealing process. The resistivity of the *a*-IGZO film covered with SiN_x_ was lower by approximately three orders of magnitude compared to that of the conventional *a*-IGZO film without any layers, and this electrical property remained unchanged even after the annealing process (Fig. 2 (SiN_x_/*a*-IGZO)). In contrast, the as-grown sample covered with SiO_x_ showed low resistivity, which increased similar to the resistivity of the conventional *a*-IGZO after the annealing process (Fig. 2 (SiO_x_/*a*-IGZO)).

Very low resistivity was observed only in the sample wherein a SiN_x_ layer formed on the *a*-IGZO film, and it was maintained regardless of the annealing. The sample wherein the conventional *a*-IGZO film and SiO_x_ were deposited on top showed a relatively high resistivity, unlike the sample wherein the SiN_x_ layer was deposited. Additionally, the resistivity tended to increase after annealing. When the SiN_x_ layer was deposited below the *a*-IGZO film instead of above it, the resistivity of the *a*-IGZO film was similar to that of the conventional *a*-IGZO film, regardless of the annealing process (Fig. 2 (*a*-IGZO/SiN_x_), (SiN_x_/*a*-IGZO/SiN_x_), and (SiO_x_/*a*-IGZO/SiN_x_)). Thus, the SiN_x_ layer deposition under the *a*-IGZO film did not significantly affect its resistivity, regardless of the annealing process. The SiOx/IGZO layer formed on the SiNx showed a relatively low resistance compared to the SiOx/IGZO formed on the thermal SiOx, but a higher resistance was still observed compared to the sample formed on the SiNx. Resistivity was strongly affected during the formation of SiN_x_ or SiO_x_ layer on the *a*-IGZO film. According to previous studies^25^, the injection of hydrogen as a shallow donor induces an increase in electrical conductivity and enables hydrogen to diffuse deeper into the bulk *a*-IGZO film through the annealing process^26^. Therefore, demonstrating the relationship between the amount of hydrogen and the annealing process and determining the mechanism behind the resistivity variation is necessary.

**Figure 3:**
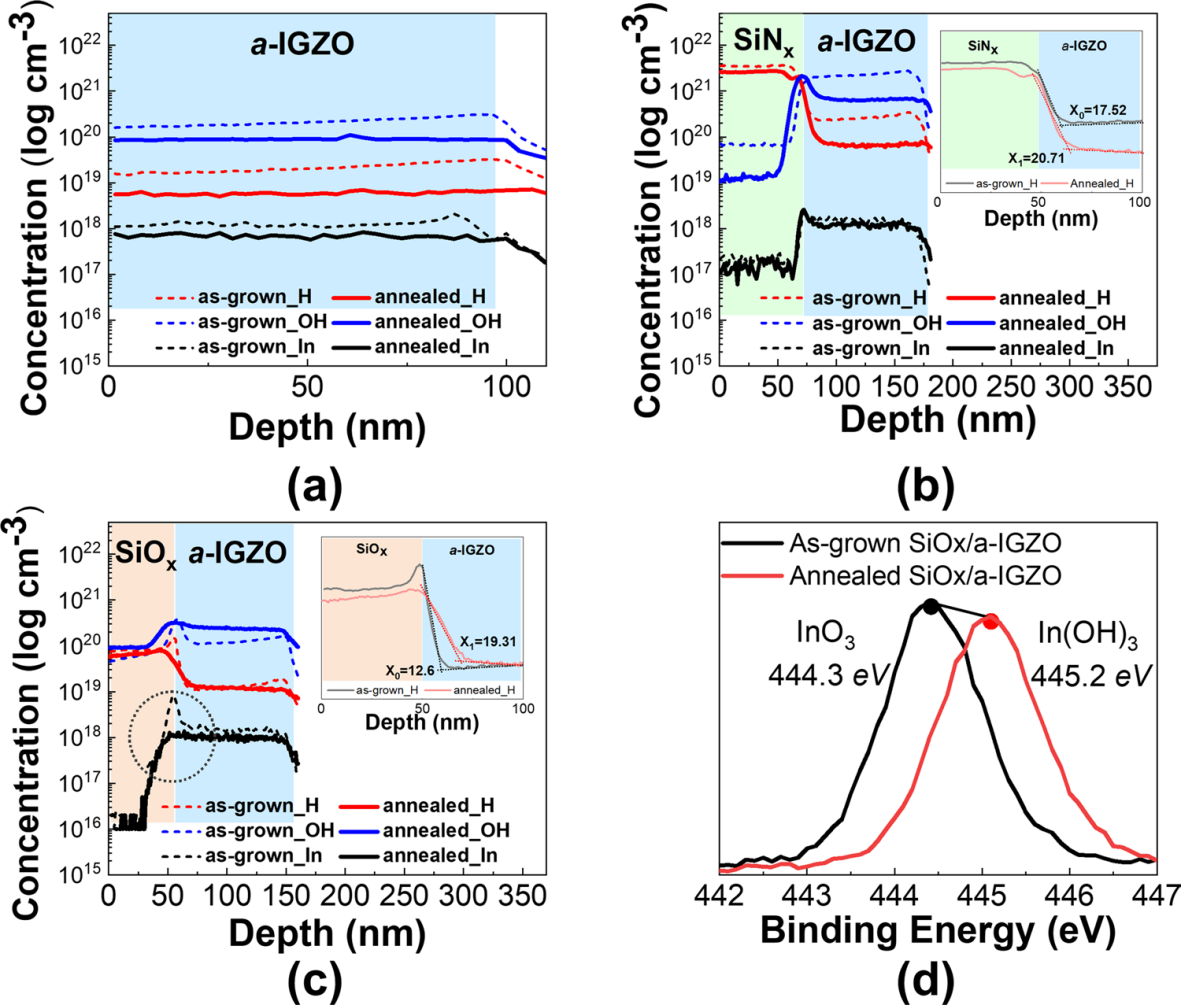
SIMS depth profiles of hydrogen, hydroxide (OH), and indium (In) in as-grown and annealed *a*-IGZO films; (**a**) conventional *a*-IGZO film, (**b**) *a*-IGZO film covered with SiN_x_, (**c**) *a*-IGZO film covered with SiO_x_, and (**d**) XPS spectra of the In3d intensity in the dashed open circle area shown in (**c**) for as-grown and annealed *a*-IGZO films covered with SiO_x_.

Figure 2 shows that the electrical properties of the *a*-IGZO films change sensitively based on the material deposited on top of the *a*-IGZO layer. To confirm the effect of hydrogen or oxygen, material analysis was performed only on the structure wherein the material was deposited on the upper part of the *a*-IGZO film, where the electrical properties were sensitive. Because a hydrogen atom is exceptionally small, identifying the exact location or content of hydrogen is not easy. In this study, the hydrogen concentration in the interior and interface of the layer was measured via SIMS, which can efficiently analyze small atoms. Figure 3 shows the SIMS depth profile analyses for hydrogen (H), hydroxyl oxygen (O–H), and indium (indium was monitored for determining the boundaries of *a*-IGZO films). The as-grown *a*-IGZO film quantitatively contained ~ 10^19^ cm^−3^ of hydrogen and a slightly inclined shape was observed at the interface with the SiO_2_/Si substrate, as shown in Fig. 3a. The amount of hydrogen and O–H decreased slightly after the annealing process, and the inclined shape became flat because the structure of the atoms in the *a*-IGZO film deposited via sputtering was stabilized through the annealing process. In Fig. 3b, it can be observed that the measured hydrogen and O–H concentrations in the *a*-IGZO film covered with SiN_x_ were one order of magnitude greater than that in the conventional *a*-IGZO film. The increase in the amount of hydrogen inside the *a*-IGZO film can be attributed to two reasons. The first is that hydrogen permeates from the *a*-IGZO layer surface owing to the hydrogen plasma generated in the SiN_x_ layer during the PECVD process by which the number of hydrogen atoms increases. The second is that the large amount of hydrogen contained in SiN_x_ migrates into the *a*-IGZO layer after the formation of the SiN_x_ layer. The observed low resistivity was due to the infused hydrogen functioning as a shallow donor, and the vacancies generated by the plasma^27^. After the annealing process, the amounts of both hydrogen and O–H were reduced; however, a significant amount of hydrogen and O–H were present in the *a*-IGZO film, which was higher than that in the conventional *a*-IGZO film. Because a large amount of hydrogen was physically injected by the plasma, the measured hydrogen content on the surface of the as-grown *a*-IGZO film covered with SiN_x_ was ~ 10^20^ cm^−3^, which was one order of magnitude higher than that in the conventional *a*-IGZO film. The injected hydrogen diffused deeper during annealing, as shown in the inset of Fig. 3b. The hydrogen and O–H profiles reduced exponentially with depth. After the annealing process, the amount of hydrogen was approximately half an order lower than that in the near-surface region, and the diffusion depth was increased. The diffusion coefficient was obtained using the least-squares fit of the hydrogen SIMS depth profile to a complementary error function as follows:

1 $$C_{D} \left( {x,y} \right) = C_{o} erfc\left( {\frac{x}{{2\sqrt {Dt_{D} } }}} \right),$$

where, ${C}_{0}$ denotes the amount of hydrogen on the surface, $D$ is the diffusion coefficient, ${t}_{D}$ denotes the annealing time, and $x$ denotes the diffusion depth of hydrogen^28^. Hydrogen diffusion depths of 19.52 nm and 22.71 nm were obtained for the as-grown and annealed *a*-IGZO sample covered with SiN_x_, respectively. As shown in Fig. 2, the electrical properties of the *a*-IGZO sample covered with SiN_x_ had very low resistance regardless of the annealing process. SiN_x_ may cause a critical hydrogen *n*-doping phenomenon in a transistor using *a*-IGZO as the active layer because of its high hydrogen concentration and hydrogen diffusion depth of 20 nm or more.

In contrast, the as-grown *a*-IGZO film covered with SiO_x_ contained a slightly lower amount of hydrogen than the *a*-IGZO film covered with SiN_x_. The hydrogen in the SiO_x_ could not move or be injected smoothly into the *a*-IGZO film, as shown in Fig. 3c. The concentrations of indium, hydrogen, and O–H increased rapidly at the interface between the SiO_x_ and *a*-IGZO layers of the as-grown samples. This imbalance is presumably caused by the unstable distribution of atoms and the increase in the element densities at the interface, and disappeared by annealing. Notably, changes were observed in the hydrogen and O–H concentrations after annealing. The amount of hydrogen was changed negligibly, whereas the amount of O–H increased in the *a*-IGZO film covered with SiO_x_. Indium-out diffusion (dished open circle in Fig. 3c) was unusually observed at the interface between the SiO_x_ and *a*-IGZO layers, which is presumably related to the increase in the O–H concentration. This phenomenon was not observed in the *a*-IGZO film covered with SiN_x_. To confirm this phenomenon, XPS measurements were performed for the oxidation state of indium (In3d_5/2_), as shown in Fig. 3d. Generally, indium exists in the form of InO_3_ in an *a*-IGZO host matrix. In Fig. 3d, indium in the as-grown state or before the annealing process forms a stable state by combining with oxygen. The InO_3_ peak at the binding energy of 444.6 eV shifts to a In(OH)_3_ peak at the binding energy of 445.2 eV through annealing. This is the indium surface oxidation phenomenon of *a*-IGZO films caused by N_2_O plasma, which is a precursor of the SiO_x_ layer used during the SiO_x_ PECVD process. As SiO_x_ is generated on the *a*-IGZO film, In(OH)_3_ is presumably formed owing to the continuous oxidation of indium at the interface and bonding with the hydrogen in SiO_x_. SIMS measurements confirmed that In(OH)_3_ out-diffused into SiO_x_. As the bonding states of indium, hydrogen, and oxygen changed, the resistivity of the *a*-IGZO film covered with SiO_x_ increased.

**Figure 4:**
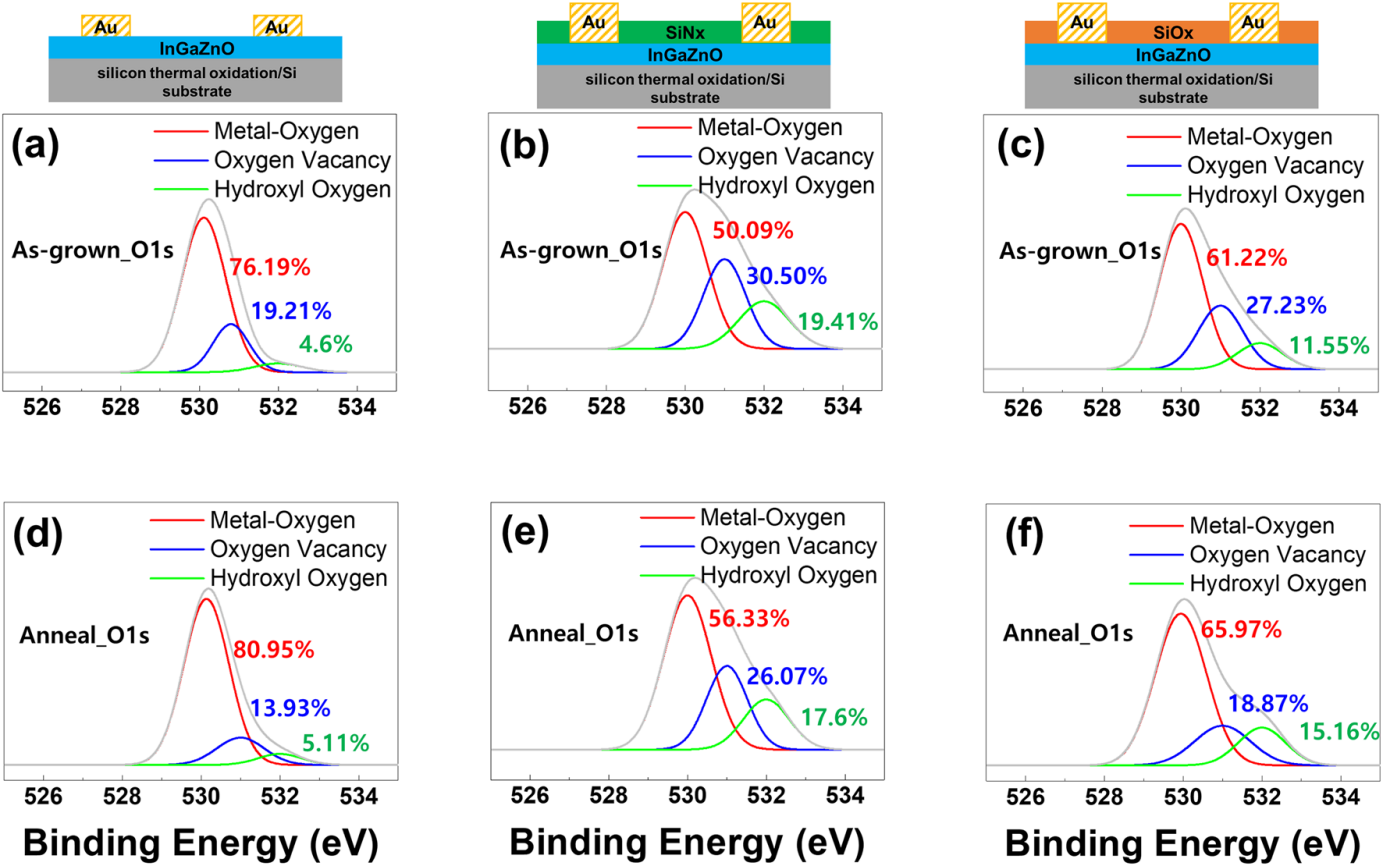
XPS deconvolutions of O1s intensities in *a*-IGZO films; (**a**) as-grown *a*-IGZO film, (**b**) as-grown conventional *a*-IGZO film covered with SiN_x_, (**c**) as-grown *a*-IGZO film covered with SiO_x_, (**d**) annealed conventional *a*-IGZO film, (**e**) annealed *a*-IGZO film covered with SiN_x_, and (**f**) annealed *a*-IGZO film covered with SiO_x_.

The change in the bonding state according to the oxygen-based SiO_x_ material and the oxygen-free SiN_x_ material formed on the *a*-IGZO could have an extremely sensitive effect on the electrical characteristics of the tens of nanometers of the oxide semiconductor. To confirm the specific binding state, the interfacial effect of thermal energy injection on the three different device structures was studied through XPS measurements. Figure 4 shows the XPS deconvolutions of the O1s intensities in the *a*-IGZO films. The three peaks are attributed to the metal–oxygen (M–O) bonds at 530 eV, oxygen vacancies (V_o_) at 531 eV, and hydroxyl (O–H) bonds at 532 eV^19^. Figure 4a,b,c show the XPS signals of three different *a*-IGZO device structures (as-grown state). The concentration of the oxygen vacancies in *a*-IGZO layers covered with SiN_x_ and SiO_x_ were observed to be higher than that in the conventional *a*-IGZO. The deposition of additional material on top of the *a*-IGZO layer changes the *a*-IGZO interface state. The signal at 530 eV, indicating the M–O state, tends to reduce gradually as SiN_x_ or SiO_x_ is deposited on the *a*-IGZO layer, resulting in a relative increase in the oxygen defect states or hydroxyl groups. It could be physically the oxygen vacancy generation via the plasma bombardment of the precursor gases during the PECVD deposition of SiN_x_ or SiO_x_ stacks. These vacancies generate free electrons in the *a-*IGZO layer^29^, resulting in low resistivity. The reduction in oxygen vacancies and O–H bonds by reducing the damage caused by the plasma bombardment during annealing was observed, as shown in Fig. 4. Figures 4d,e,f show the deconvoluted O1s peaks of the annealed structures, wherein an elevated O–H bond concentration can be observed. The concentration of O–H bonds in Fig. 4f increased from 11.55% to 15.16%, similar to that observed in the SIMS measurement data (Fig. 3c). Oxygen vacancies and O–H bonds, which are normally considered defects in the host matrix, were reduced by supplying thermal energy. In the case of the *a*-IGZO film covered with SiO_x_, the O–H bonds increased when thermal energy was supplied. The following section discusses the correlation between the change in the host matrix and electrical resistance caused by the movement of hydrogen atoms.

**Figure 5:**
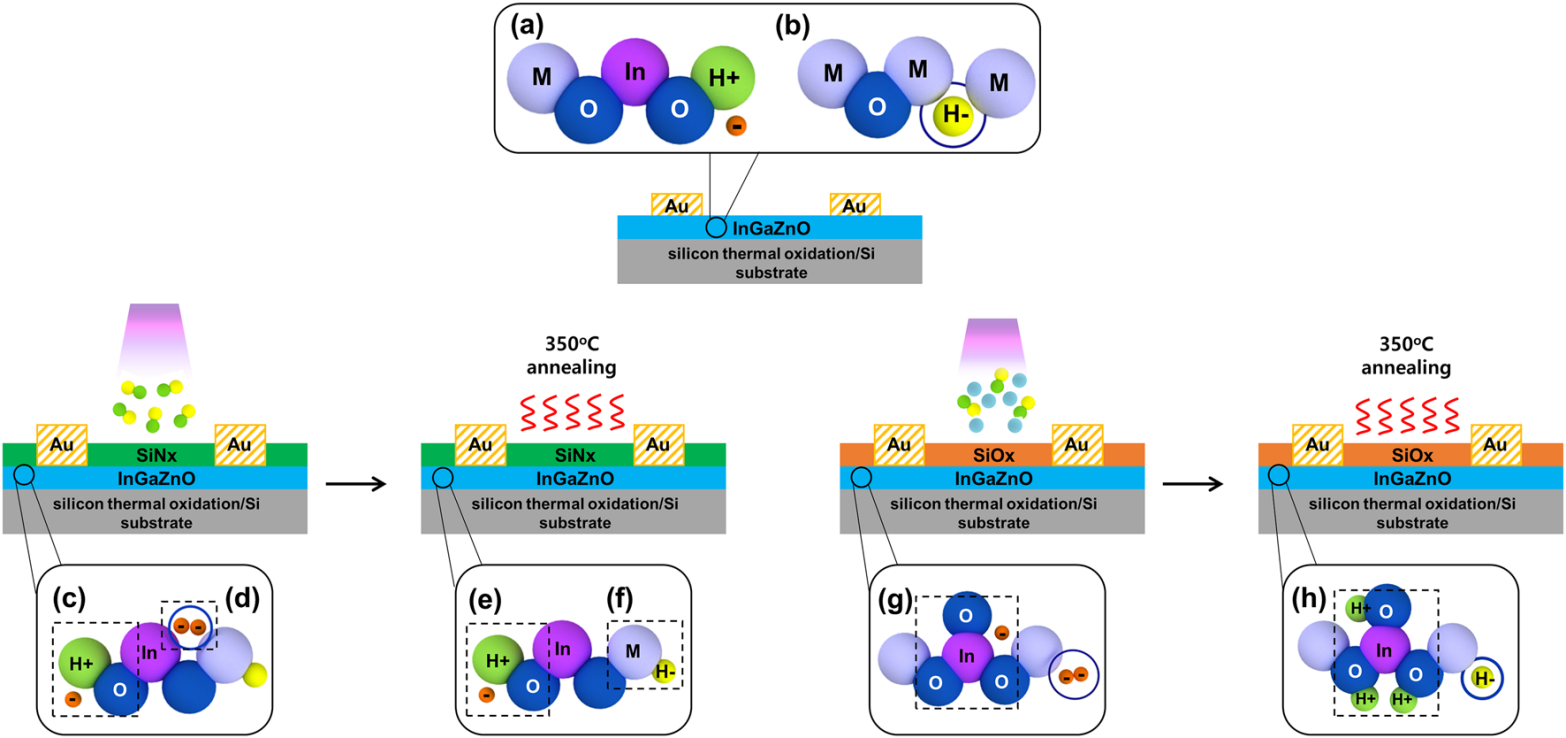
Schematics of (**a**) shallow donor generation in conventional *a*-IGZO film, (**b**) passivation of defects by hydrogen in conventional *a*-IGZO film. Formation of hydrogen, oxygen, and indium-related states (**c**)–(**f**) in *a*-IGZO films covered with SiN_x_, and (**g**)–(**h**) in *a*-IGZO films covered with SiO_x_.

In conventional *a*-IGZO films, hydrogen can be found in the H_0_, H^+^, and H^−^ states. The existence of these three different states of hydrogen in an *a*-IGZO film depends on the host matrix state and surrounding environment of the hydrogen atom. Interestingly, hydrogen atoms replace oxygen vacancies in oxide-based materials, acting like oxygen; these atoms can then combine with metals^25^. This phenomenon mainly occurs when the hydrogen in the H^−^ state either makes the defect states generated by oxygen vacancies disappear or moves below the Fermi level to deactivate the defect state^30^, resulting in the formation of thermally stable metal–hydrogen bonds (M–H), as shown in Fig. 5a. In contrast, hydrogen in the H^+^ state reacts with O^2−^ and donates an electron via the following reaction shown in Fig. 5b:

2 $${\text{H}}^{ + } + {\text{O}}^{2 - } \to {\text{OH}}^{ - } + {\text{e}}^{ - }$$

When an *a*-IGZO film contains hydrogen at [H] ≫ 10^20^ cm^−3^, most of the hydrogen atoms form O–H bonds rather than H_2_ or H_0_ molecules^31^. Ionized H^+^ acts as a shallow donor by releasing free electrons through bonding with oxygen, as shown in Eq. (2). In the case of SiN_x_ deposited on top of the *a*-IGZO film, the supply of large quantities of hydrogen from SiN_x_ rapidly increases the hydrogen content in the *a*-IGZO film and changes the environment such that hydrogen and oxygen can be easily combined, as shown in Fig. 5c. One of the origins of the free electrons is the increasing number of shallow donors by the plasma treatment of SiH_4_ and NH_3_ gas, which are SiN_x_ precursor gases. Another reason is the bombardment damage to the upper surface of *a*-IGZO generated during the plasma process (Fig. 5d). The bombardment damage causes oxygen to escape or change its location, thereby inducing oxygen vacancies. Therefore, the as-grown *a*-IGZO film covered with SiN_x_ exhibited a low resistivity and high oxygen vacancy concentration. During annealing at 350 °C, the weak O–H bonds are easily deformed and migrated by the thermal energy, thereby decreasing the O–H concentration, as confirmed by the SIMS and XPS results. However, the *a*-IGZO covered with SiN_x_ still contained a large amount of hydrogen because a large amount of hydrogen was already infused into the *a*-IGZO film through the PECVD process, and thermal energy did not affect the hydrogen combined with the metal (H^−^), as shown in Fig. 5e,f. This causes a low resistivity in the *a*-IGZO film covered with SiN_x_ after annealing.

In contrast, Fig. 5g shows that InO_x_ in the *a*-IGZO film readily reacts at the interface between SiO_x_ and *a*-IGZO film with the oxygen precursor (N_2_O gas) injected during SiO_x_ deposition. This reaction generates InO_3_ by combining InO_2_ with the excess oxygen supplied by the capture of surrounding free electrons^31^. As confirmed from the SIMS measurements, the indium peak diffused outward from the *a*-IGZO layer to the SiO_x_ layer, and the hydrogen and O–H peaks rapidly increased at the interface between SiO_x_ and *a*-IGZO. When thermal energy is supplied to the *a*-IGZO film covered with SiO_x_, InO_3_ captures the surrounding free electrons and combines with ionized hydrogen to form In(OH)_3_^32^, as shown in Fig. 5h:

3 $${\text{InO}}_{{\text{x}}} + {\text{H}}^{ + } + {\text{e}}^{ - } \to {\text{In}}\left( {{\text{OH}}} \right)_{{\text{x}}}$$

Additionally, ionized hydrogen (H^−^) moves to the vacancy site by supplying thermal energy, which results in vacancy passivation and decreases the number of free electrons. This reaction can be confirmed from the XPS spectra of In3d shown in Fig. 3d and the SIMS results shown in Fig. 3c, wherein the O–H concentration can be observed to increase. Based on the aforementioned processes, the resistivity of the sample covered with SiO_x_ increased.

## Conclusion

We studied the correlation between the hydrogen behavior and electrical properties of oxide semiconductor devices. The test samples were fabricated with six different types of structures, and the electrical properties of the *a*-IGZO films were observed to change sensitively depending on the material formed on the upper part of the oxide semiconductor. The dynamic increase and decrease in the electrical resistivity of an *a*-IGZO semiconductor is confirmed to be closely correlated with the amount of hydrogen present in the two insulators used as the passivation and gate insulator layers in the semiconductor process. In the case of the SiN_x_ layer, a high hydrogen content (8 × 10^19^ cm^−3^) was confirmed, and the hydrogen concentration at the interface was extremely high because of the hydrogen injected into the *a*-IGZO layer during the SiN_x_ PECVD process. Moreover, a hydrogen diffusion length of 22 nm or more was confirmed to have formed through the annealing process. In contrast, In(OH)_3_ was formed at the interface and out-diffused to SiO_x_ in the *a*-IGZO layer, and the concentration (3 × 10^20^ cm^−3^) of O–H was significantly higher than that of hydrogen after annealing.

Hydrogen is always present in oxide semiconductors and is affected by various factors, such as the state of bonding with metal or oxygen and the vacancies of oxygen. To use oxide semiconductors for various applications, understanding the hydrogen behavior is essential, and additional research is required on methods to control the hydrogen content and functions in oxide semiconductors such that it can function as desired.

## Data Availability

The datasets generated during and/or analyzed during the current study are available from the corresponding author on reasonable request.
